# More is different: how aggregation turns on the light

**DOI:** 10.1093/nsr/nwaa266

**Published:** 2020-11-28

**Authors:** Philip Ball

**Affiliations:** Writes for NSR from London

## Abstract

The reductionist approach to science seeks to understand the behaviour of systems by studying their individual components. It has been an enormously productive approach, but it is also widely acknowledged now that in some systems the behaviour of interest is an emergent property that cannot be discerned in the separate parts. Biology is replete with such examples, from the flocking of birds to the way metabolic processes in cells rely on a dynamic interplay of proteins and other components.

Yet molecular systems do not have to be particularly complex before their properties become more than the sum of the parts. A classic example is the appearance of bulk-like metallic behaviour in small clusters of metal atoms only once they exceed a certain critical size. One of the most striking instances became apparent in 2001, when Ben Zhong Tang of the Hong Kong University of Science and Technology and his co-workers found that heterocyclic silicon-containing molecules called siloles become luminescent as nanoscopic aggregates even though the individual molecules in dilute solution do not emit light [1]. This looked like the opposite of the well-known phenomenon of concentration quenching, in which energy transfer between fluorescent (generally organic) molecules quenches the emission, an effect explained in 1955 [2]. Aggregation-induced ‘switching off’ is intuitively understandable, but ‘switching on’ due to aggregation was more surprising.

Yet this effect of ‘aggregation-induced emission’ (AIE), as Tang and colleagues called it, was apparently seen, but not understood, much earlier [3]. In the 1850s, George Stokes noted that some inorganic complexes were fluorescent in the condensed, solid state but not in solution. At first, AIE was seen as a curiosity and deemed likely to be rare. However, subsequent research has shown not only that it is a rather common effect but also that it can be considered just one manifestation of a wide range of behaviours that arise from aggregation—leading to the proposed field of ‘aggregate science’, manifesting at the supramolecular level of small clusters or groups of molecules held together by relatively weak interactions. The field might be considered to illustrate George Whitesides’ notion of a chemistry ‘beyond the molecule’ [4], which bridges disciplines ranging from colloid science to crystal growth, nanotechnology, liquid crystals, photochemistry and molecular biology. At the same time, it echoes the famous insight of physicist Philip Anderson about emergent phenomena and the hierarchical nature of science: ‘More is different’ [5]. An ability to switch properties on and off by controlling intermolecular interactions and aggregation suggests various applications, from optical device technologies to targeted drugs for cancer therapy [6].

NSR spoke to Ben Zhong Tang about the origins and possibilities of the field.

***NSR*:** It seems you noticed AIE in 2001 by accident. How did it come about?

***Tang*:** Yes, it was serendipity. Development of new light emitters for the fabrication of organic light-emitting diodes was a hot topic at that time. We were trying to make new luminophores [light-emitting molecules] with high efficiencies and novel structures. Attracted by the aesthetically pleasing molecular structures of siloles, I asked my students to prepare various silole compounds. One day, a student told me that he could not see any luminescence when he used a UV lamp to excite the solution of the silole compound he had made. This surprised me, because I myself prepared a silole compound when I was a PhD student and I remember that its crystal was luminescent. I sensed something strange and immediately rushed to the lab. After careful verification and discussion with the student, we concluded that both of us were correct: the silole solution was not luminescent (his observation was right) but the silole powder was emissive (my memory was right). The non-luminescent molecular species in the dilute solution were induced to emit light through formation of aggregates in the solid state. We termed the process aggregation-induced emission or AIE.

A mesoscopic aggregate can have a property that its molecular species does not exhibit at all.

—Ben Zhong Tang

**Figure:**
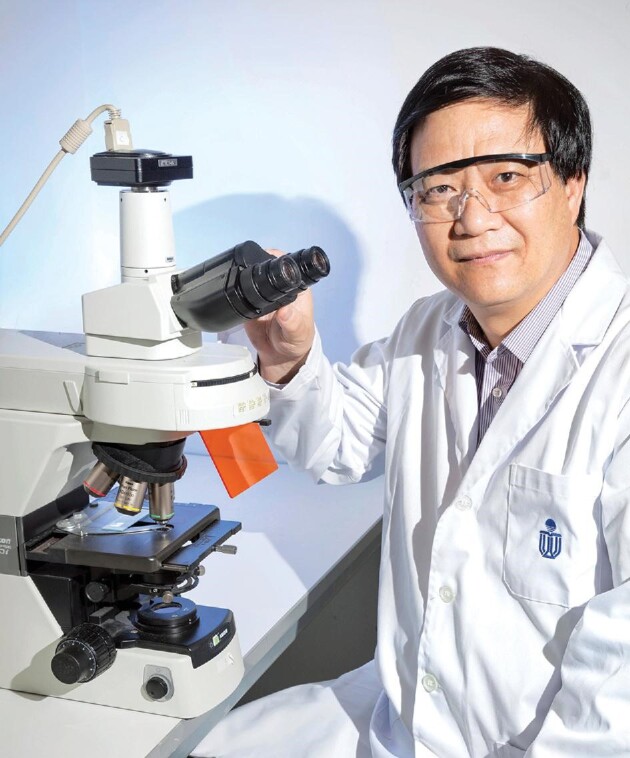
Ben Zhong Tang of the Hong Kong University of Science and Technology, China (*Courtesy of Ben Zhong Tang*).

***NSR*:** The phenomenon seemed to defy conventional expectations. Did you have trouble persuading others—or yourselves!—that it was real?

***Tang*:** I initially thought the student might have done something wrong, for the phenomenon he observed was totally unexpected. The common belief in the community of photophysics research is that luminescence from an organic dye generally weakens when its molecules are aggregated, an effect often referred to as aggregation-caused quenching or ACQ. I was shocked when I realized that the silole luminogen was showing an anti-ACQ effect. Still, I felt lucky to encounter something ‘abnormal’. No matter how odd a phenomenon seems, if it can be repeatedly observed, it must be real. We repeated our experiments many times and we were eventually convinced that the AIE effect was true. We had trouble, however, to understand why the silole luminogen behaved in such a way that was diametrically opposed to conventional ACQ.

***NSR*:** Are there any historical precedents—experiments in which this effect might have been glimpsed previously, but not recognized as such?

***Tang*:** When we published our first AIE paper in 2001, we thought the photophysical effect was unprecedented. However, we gradually found out that similar phenomena had been previously observed by other scientists. For example, in 1853 George Stokes reported in a paper that some inorganic platinocyanide salts ‘are *sensitive*’ (meaning *luminescent* in modern terminology) ‘only in the solid state’ but ‘their solutions look like mere water’. Sadly, he didn’t follow it up. Other people have made similar observations in different dye systems, which were, however, not recognized as AIE processes. Partially because of this, we had great difficulty in finding relevant reference papers. As a matter of fact, Stokes’ report, published in the mid-19th century, was not known to us until the middle of 2018. However, we are not surprised by those early works, for we understand that science progresses not in an abrupt but in a continuous way. George Smith articulated this: ‘Very few research breakthroughs are novel. Virtually all of them build on what went on before.’ A discovery is often a happenstance. We happened to have ‘rediscovered’ a very old but largely unnoticed phenomenon. Luckily, we grasped the opportunity to see more and farther by standing on the shoulders of giants.

## GETTING TO GRIPS WITH AGGREGATION EFFECTS

***NSR*:** What is the origin of the phenomenon, and how did you deduce it?

***Tang*:** This AIE was truly intriguing but we had no idea at all about its origin at first. We read many papers, but nearly all of them discussed why aggregation was detrimental to luminescence through ACQ. We scrutinized the silole structures and noticed that they were conformationally flexible, containing many movable groups or units. Their dynamic intramolecular motions may have dumped the silole excitons into a dark state, making the molecules non-luminescent. We figured aggregate formation may have stiffened the silole structures and blocked their nonradiative decay channels, thus switching on their light emission.

We designed many experiments to test our hypothesis. We imposed controls on the intramolecular motions of the AIE luminogens (AIEgens) externally by physical constraints (temperature, viscosity, pressure, crystallization and so on) and internally by chemical reactions (such as cyclization, aromatization, substitution and cross-linking). The data from the control experiments supported our hypothesis that restriction of intramolecular motions (RIM) was responsible for the observed AIE phenomena. Five years later, our AIE research was extended to tetraphenylethylene (TPE) systems. While almost nobody had discussed why the silole molecules were non-luminescent as isolated species, some researchers had previously suggested that the non-emissive nature of single molecules of some TPE derivatives was due to their active intramolecular motions. The real cause for the AIE effect of TPE, however, is more complicated, and in fact we are still deciphering it.

***NSR*:** How general an effect is it? What are the features of molecules that exhibit it?

***Tang*:** At first, we thought AIE was a very special phenomenon only observable in the silole systems, but now we are fully convinced that it is a general photophysical process occurring in many luminogen systems. Thanks to the research efforts of our and other groups, thousands of fluorescent and phosphorescent AIEgens have been developed so far. Their emission colours cover the whole visible spectral range and extend well into the near-infrared; some of these species emit in quantum yields up to unity (100%). The AIEgens are very varied, spanning from organics to inorganics, from small molecules to macromolecules, from conjugated nanoparticles to nonconjugated clusters, from discrete organometallic complexes to metal–organic frameworks, from single crystals to multicomponent mixtures … No matter what type they belong to, the AIEgens all share a common structural feature: they are flexible as molecular species but rigid as aggregates or clusters.

***NSR*:** Is AIE amenable to design? Can you make molecules to order that will show it, and control the intensity, say? Is it possible to make mixtures that show the effect?

The real joy of doing scientific research is its unpredictable nature: a small, unexpected detail may completely alter its trajectory.

—Ben Zhong Tang

***Tang*:** Yes, it can be readily designed. Any luminogens consisting of dynamically moving structural units are potential candidates for AIEgens. When the luminogenic molecules are fabricated into thin films in the solid state or into tiny particles in aqueous media, their intramolecular motions are restricted, the RIM mechanism is activated, and the AIE effect is turned on. When the luminogenic molecules are crystallized, their light emission increases in intensity and is shifted in colour hypsochromically [to a shorter wavelength] in comparison to those of their amorphous counterparts, due to the stronger RIM effect imposed on the AIEgens by tighter packing in the crystals. Not only homogeneous aggregates of the same molecules but also heterogeneous mixtures of different molecules can show AIE, as the RIM mechanism is component independent. Anything that can stiffen the structures of the aggregate mixtures can activate AIE.

## LESSONS AND USES

***NSR*:** What potential applications of AIE exist, both for fundamental science and for technologies? And which of them have been realized?

***Tang*:** AIE has far-reaching fundamental and practical implications. AIE shows that a mesoscopic aggregate can have a property that its molecular species does not exhibit at all. This calls for a paradigm shift in research philosophy from reductionism to emergentism, in order to better understand processes and properties through a structural hierarchy. Bearing this in mind, we embarked on a journey to explore the processes and properties unique to aggregates. Examples include efficient room-temperature phosphorescence from pure organic aggregates, crystallization-induced light emission, aggregation-boosted generation of reactive oxygen species, anti-Kasha transitions [that is, not from the lowest excited state] from high-energy excitons, and clustering-triggered ‘clusteroluminescence’, all of which are difficult to access with isolated molecular species but are readily accessible to multimolecular aggregates. In addition to the fundamental impact on research philosophy, AIEgens are practically useful, because materials are commonly used in robust forms in the (aggregated) solid state. We have demonstrated that AIEgens are promising for an array of high-tech applications in such areas as biomedical theranostics, chemical sensing, optoelectronic devices and stimuli-responsive systems.

***NSR*:** One of the key general lessons of AIE is that the properties of a collection of molecules are not necessarily a simple extrapolation of those of the molecules in isolation. In other words, entirely new possibilities open up for supramolecular systems. Is this a paradigm that you are exploring further?

***Tang*:** Yes, we are exploring the possibility of developing aggregate mesoscience from the invaluable lessons we have learned through our AIE study. More than 2500 years ago, the Daoist sage Laozi said that ‘One begets two; two begets three; three begets ten thousand things.’ This expresses a simple yet profound philosophical principle that gradual increase (one to two to three) may lead to a sudden ‘quantum jump’ (three to ten thousand). A similar idea was expressed by Aristotle: ‘The whole is greater than the sum of its parts.’ When individual parts are clustered together to form one entity, the parts in the cluster are worth more than if the parts were separated. Although the reductionist methodology has made great contributions to scientific advance, it is powerless to cope with nonlinear complex systems, and may even shackle new ideas—discouraging one from, for example, investigating a property of a substance that its molecular components do not possess.

***NSR*:** In which directions have other groups taken this research?

***Tang*:** AIE offers nice opportunities that have attracted many groups to study it. The area is booming, as reflected by the large numbers of AIE publications in recent years: a Google Scholar search for ‘aggregation-induced emission’ shows that 4660 papers were published on the topic in 2019. Some have studied fundamental aspects of AIE, such as the processes of molecular motion and structural rigidification; some have developed novel AIE systems such as room-temperature phosphorescence, clusterization-triggered luminescence and biogenic AIEgens; others have explored potential applications of AIEgens in stimuli-responsive systems, chemical sensors, biomedical probes, optoelectronic devices and so on. These research efforts will deepen our understanding of natural processes at the aggregate level, broaden our horizons on the mesoscale and generate new mesomaterials with advanced functionalities.

***NSR*:** One might consider AIE a good example of the importance of taking seriously findings that don’t fit with expectations. Is there a general lesson in that? Can it be risky to follow up results that seem to conflict with ‘received wisdom’, or that don’t fit with the initial aims of a research project?

***Tang*:** Different people have different styles of carrying out scientific research: some are objective-oriented, some are curiosity-driven, some take an open, ‘blue skies’ view … However, few projects ever go as straightforwardly as planned; if they do, they might end up being a run-of-the-mill study. The real joy of doing scientific research is its unpredictable nature: a small, unexpected detail may completely alter the trajectory of the research. I teach my students that when they encounter an unanticipated detail, the first thing to do is to carefully examine whether the phenomenon is repeatable or the data are reproducible—it could be a good chance, or bad luck, or even a stupid mistake. If the detail turns out to be true, you need to evaluate its value: is it trivial or crucial? If it can’t be explained by mainstream paradigms, or if it conflicts with the generally received wisdom, you should follow it up—you might be standing at the doorway to a meaningful discovery. In fact, breakthrough is often serendipity. It may take painstaking effort to decipher the mysteries behind the unsought detail, but the reward is also huge: the excitement of blazing a new trail!

## THE ROUTE TO SUCCESS

***NSR*:** You now have an ‘AIE Institute’ in Guangzhou. How did that come about, and what are its aims?

***Tang*:** Two years ago, I attended a brainstorming meeting in Guangzhou where I shared my thoughts on the transformation of the Guangdong–Hong Kong–Macau Greater Bay Area into a knowledge-based community and technology-oriented society. The municipal leadership of Guangzhou City liked my ideas and gave the green light to my proposal to build an institute for translational research, aimed at bridging the gap between academia and industry. Under the auspices of Guangzhou City, Huangpu District and South China University of Technology, we built an AIE Institute located in the new town of Guangzhou Science City. The mission of the institute is to innovate in high technologies and develop new products. We hope it will become an incubator for nurturing the next generation of technologists and entrepreneurs.

***NSR*:** How do you recruit your research team or your collaborators? What qualities do you look for?

***Tang*:** When I evaluate an applicant who wishes to join my team, I give great weight to qualities like enthusiasm and passion, in addition to other factors like talent and knowledge. The path to scientific discovery may never be straightforward; research is like mountaineering or rock climbing, which requires burning zeal. Karl Marx put it vividly: ‘There is no royal road to science, and only those who do not dread the fatiguing climb of its steep paths have a chance of gaining its luminous summits.’ Self-motivated people will not be overwhelmed by the obstacles on the road, but will enjoy the processes of problem solving. Positive behaviour is contagious, and an optimistic attitude helps build a healthy lab culture and a buoyant working environment. When a lab is full of passionate team players, the harmonic working mood will help enhance its chance to make new inventions. Scientific research has become more and more complex, and many issues and problems can only be tackled through collaborative effort. I have collaborated with many people over the years, and many of these collaborations were built through conference discussions and campus seminars. Our AIE research program has developed into various disciplines in science, engineering and biomedicine, largely thanks to our collaborations with colleagues with different backgrounds but complementary expertise.

***NSR*:** What have been your influences or inspirations in developing your career?

***Tang*:** I was taught by different education systems: I received my BS and PhD degrees from Chinese and Japanese universities, respectively, and conducted my postdoctoral study at a Canadian university. I was thus trained by both Eastern and Western research philosophies. I tried to take the best aspects of both worlds to run my research lab when I commenced my independent academic career at the Hong Kong University of Science and Technology. I was reminded by the senior faculty there to work together with other people, because everything in Hong Kong is small in scale: tiny labs, few students, little money … If one wants to do something big on this small island, one must team up with other colleagues. The slogan is: ‘If you want to go fast, go alone; if you want to go far, go together.’ I was much influenced by this notion, and actively sought for research collaborations through the years. The spirit I am advocating now in the AIE research community is ‘United we stand, together we shine!’ I am trying to create a win–win situation: to further develop my own career and meanwhile help others, particularly young people, to grow.

***NSR*:** What would your advice be to young researchers today?

***Tang*:** A critical decision a scientist must make is to pick a ‘right’ project. This is especially true for a young researcher. Everyone wishes to do original research and dreams of making an impactful breakthrough. However, this is easier said than done. Science has advanced to such an extent that almost nothing has not been touched by someone in the past. So, it's impossible to start anything from scratch. Science, like anything else, evolves continuously, as Parmenides said: ‘Nothing comes from nothing.’ So, we should not be ashamed of doing something people have done before; after all, it's beyond anyone's capability to know all the previous studies. Sure, we may occasionally reinvent the wheel, particularly one that has been overlooked or forgotten by the research community. However, we must strive to find new things from the old stuff through our research, or ‘to gain new insights by studying the past’, as Confucius taught. The Nobel laureate Albert Szent-Györgyi said that ‘research is to see what everybody else has seen and to think what nobody else has thought.’ Clearly, critical thinking is of crucial importance to scientific research. Remixing various old things in a thoughtful way can lead to new discoveries and breakthroughs. Let me finish by quoting Steven Jobs’ famous saying: ‘We’re not going to be the first to this party, but we’re going to be the best!’
